# Studying Mammary Physiology and Pathology in Domestic Species Benefits Both Humans and Animals

**DOI:** 10.1007/s10911-023-09547-9

**Published:** 2023-07-14

**Authors:** Katherine Hughes

**Affiliations:** Department of Veterinary Medicine, University of Cambridge, Madingley Road, Cambridge, CB3 0ES UK

**Keywords:** Cow, Development, Dog, Goat, Mammary gland, One Health, Tumor

In 2010 the worldwide population of domesticated ruminants was estimated to be 3.6 billion [1]. Many of these animals are used for milk production or as suckler animals where the offspring are consumed for meat. These functions are dependent on the lactational capacity of the female ruminant, thus postnatal mammary development impacts future lactational productivity [2, 3] whilst mastitis is a major welfare issue that also causes financial losses [4].

In contrast to domesticated ruminants, in many cultures pets increasingly fulfil an emotional role similar to that of children, with commensurate emotional and financial investment in their care. In a survey of sixty geographically, culturally, and linguistically different societies, the most frequently mentioned pet species was the dog [5]. Mammary pathology is a major clinical issue in pet carnivores, and mammary tumours are the most frequently diagnosed neoplasm in unneutered female canines [6].

Research focused on the mammary physiology and pathology of domestic species kept by humans therefore clearly has the potential to directly benefit the species concerned, and there are also wider advantages to conducting such research studies, when viewed with a One Health lens [4]. For example, domestic species constitute non-traditional models of mammary gland development [3, 7, 8]. In addition, domestic pets and their human carers frequently share the same environment and therefore studying mammary tumours arising in pets may provide insights relevant to understanding the pathogenesis of breast cancer [9, 10].

Against this tableau of human-animal interaction, and succeeding two recent highly successful Special Issues [11, 12], I am proud to present the Journal of Mammary Gland Biology and Neoplasia Special Issue focused on Mammary Physiology and Pathology in Domestic Species.

As alluded to above, understanding mammary development underpins efforts to maximise the lactational potential of dairy ruminants [2, 3]. In this issue, Vang and colleagues employ ultrasound imaging and ultrasound-guided mammary biopsy to track udder development in a cohort of cows between ten and fifty-two weeks of age, revealing new insights into mammary development during this critical postnatal period. In addition to describing findings of relevance to the dairy industry, the authors also highlight how non-invasive imaging modalities may be employed to evaluate breast tissue during investigations focusing on human lactation [13].

In parallel to in vivo studies, in vitro approaches offer unrivalled opportunities to manipulate factors of interest within a tractable system. Kobayashi describes the biology and dysfunction of mammary epithelial tight junctions and provides a detailed protocol describing culture of mammary epithelial cells in a manner that is compatible with milk production. This article also touches upon some of the challenges that may be encountered when working with non-traditional species, such as the fact that the optimal culture conditions for mammary epithelial cells may be species-specific [14].

Using a combination of in vivo and in vitro methodologies, Tsugami and colleagues demonstrate that, in goats, the branched chain amino acid valine causes mammary upregulation of selected antimicrobial components [15, 16]. This discovery and others like it [17] may pave the way to harnessing synthesis of antimicrobial components as a therapeutic approach in cases of mastitis, an exciting prospect given the threat posed by antimicrobial resistance in both animal and human pathogens.

On the companion animal side, in this Special Issue, Edmunds and colleagues demonstrate that canine breed, age and neuter status impact the odds of being diagnosed with a mammary lesion of epithelial origin, whether a case of mammary epithelial neoplasia presents with only a single mammary epithelial neoplastic lesion or with multiple lesions, and with whether benign or malignant disease is diagnosed. By identifying previously unrecognised epidemiological patterns, the authors suggest that canine genetics impact the development of benign or malignant disease, tumour number and cellular composition, and thus shape tumour heterogeneity [18].

Using data originally derived from the study of metastasis in breast cancer, and thereby illustrating the bi-directional nature of a One Health viewpoint, Seitz and colleagues identify that reduction in expression of the Wnt-antagonist *Sfrp1* is significantly associated with metastasis formation in canine mammary cancer. They suggest that SFRP1 is a potential tumour suppressor gene as well as being a biomarker that may aid in identification of metastatic canine mammary tumours [19].

Also focusing on mechanisms of metastasis, Ettlin and colleagues use laser-capture microdissection to examine cancer-associated stroma from metastatic and non-metastatic canine mammary tumours for differentially expressed genes. They outline a panel of targets that are specifically upregulated in metastatic cancer-associated stroma and therefore likely to be implicated in malignancy and metastasis of these tumours, drawing comparisons with data regarding expression of these proteins in breast cancer [20].

Taken together, this collection of articles represents a fascinating and illuminating insight into the mammary physiology and pathology of domestic species. I hope that the reader enjoys exploring this collection.
